# A High Immune-Related Index with the Suppression of cGAS-STING Pathway is a Key Determinant to Herceptin Resistance in HER2+ Breast Cancer

**DOI:** 10.7150/ijbs.94868

**Published:** 2024-06-17

**Authors:** Ruizhao Cai, Qingshan Chen, Dechang Zhao, Yan Wang, Ling Zhou, Kaiming Zhang, Jialu Shan, Zhiling Li, Yuhong Chen, Hailiang Zhang, Gongkan Feng, Xiaofeng Zhu, Rong Deng, Jun Tang

**Affiliations:** 1 Department of Breast Oncology, Sun Yat-sen University Cancer Center, Guangzhou, China.; 2 State Key Laboratory of Oncology in South China, Guangdong Provincial Clinical Research Center for Cancer, Sun Yat-sen University Cancer Center, Guangzhou, China.

**Keywords:** Herceptin-resistance, HER2+ breast cancer, prognostic signature

## Abstract

Resistance to HER2-targeted therapy is the major cause of treatment failure in patients with HER2+ breast cancer (BC). Given the key role of immune microenvironment in tumor development, there is a lack of an ideal prognostic model that fully accounts for immune infiltration. In this study, WGCNA analysis was performed to discover the relationship between immune-related signaling and prognosis of HER2+ BC. After Herceptin-resistant BC cell lines established, transcriptional profiles of resistant cell line and RNA-sequencing data from GSE76360 cohort were analyzed for candidate genes. 85 samples of HER2+ BC from TCGA database were analyzed by the Cox regression, XGBoost and Lasso algorithm to generalize a credible immune-related prognostic index (IRPI). Correlations between the IRPI signature and tumor microenvironment were further analyzed by multiple algorithms, including single-cell RNA sequencing data analysis. Patients with high IRPI had suppressive tumor immune microenvironment and worse prognosis. The suppression of type I interferon signaling indicated by the IRPI in Herceptin-resistant HER2+ BC was validated. And we elucidated that the suppression of cGAS-STING pathway is the key determinant underlying immune escape in Herceptin-resistant BC with high IRPI. A combination of STING agonist and DS-8201 could serve as a new strategy for Herceptin-resistant HER2+ BC.

## Introduction

Breast cancer (BC) is the most common malignancy among women worldwide, and HER2+ BC constitutes 15-20% of newly diagnosed BC 1, 2. The current anti-HER2 treatments such as trastuzumab (Herceptin) and pertuzumab, have been well established for HER2+ BC and achieved a high response rate. Most recently, trastuzumab deruxtecan (DS-8201), a novel HER2-targeting antibody-drug conjugate with a novel DNA topoisomerase I inhibitor, demonstrated a significant improvement in overall survival (OS) and progression-free survival (PFS) in HER2+ metastatic BC previously treated with Herceptin, confirming as the standard of care in the second-line setting 3, 4. Although the effective HER2-targeted treatments have radically reduced the risk of recurrence and improved the survival of HER2+ BC patients, a significant proportion of patients ultimately develop resistance to these therapies, largely because of tumor heterogeneity 5-7. Thus, identification of biomarkers of response beyond HER2 status is highly needed 8. Multiple biomarkers for prognosis of HER2+ BC have been reported 8-10. Katherine *et al.* developed a proteomic classifier measured via conventional immunohistochemistry to enable the stratification of sensitive tumors early during neoadjuvant anti-HER2 therapy 8. Ziteng *et al.* constructed a pan-cancer predictive index of anti-HER2 therapies across 33 tumor types, including breast cancer, which highlighted the importance of incorporating transcriptional patterns into the assessment of HER2 status for better patient selection 9. However, these predictive models do not fully consider the impact of immune infiltration on prognosis, as a growing amount of evidence supports the key role of tumor immune microenvironment in development of HER2+ BC 11-13. Indeed, more than 50% of HER2+ BC showed a feature of “cold” tumor, displaying limited tumor-infiltrating lymphocytes (TILs) in tumor microenvironment (TME) 14. It has been proved that the higher infiltration of natural killer (NK) and CD8+ T cells can improve the tumor responses to HER2-targeted therapy by mediating direct cytotoxicity to HER2+ BC cells 15, 16. Therefore, taking immune infiltration into assessment would improve our ability to discover additional features associated with response to HER2-targeted therapy.

The innate immune system plays an important role in anti-tumor processes 17, 18. Type I interferons (IFNs), including 13 IFN-alpha, IFN-beta and many other subtypes, may be the most potent and broadly active cytokine family in innate immunity 19. A byproduct of cancer development is the accumulation of damage-associated molecular patterns that can effectively engage pattern-recognition receptors (PRRs) and activate innate immune responses 18, 19. Although various PRRs can function in the context of cancer, the recognition of aberrant DNA by the cytoplasmic protein cyclic GMP-AMP (cGAMP) synthase (cGAS) have yielded new insights into the activation of innate immune responses 19. The release of double-stranded DNA (dsDNA) from mitochondria or nucleus into the cytosol allows cGAS to catalyze ATP and GTP into cGAMP 20. Then cGAMP activates STING at the endoplasmic reticulum, in which STING undergoes an oligomerization to form tetramers and translocate to the Golgi apparatus 21. At the Golgi, the activated STING recruits TANK binding kinase 1 (TBK1) and IFN regulatory factor 3 (IRF3) for activation, at which point IRF3 translocates to the nucleus and exerts its transcriptional function in expressing type I IFN and hundreds of downstream IFN-stimulated genes (ISGs) 22, 23. The activation of the cGAS-STING pathway can not only induce death of cancer cells 22, but also promote anti-tumor immunity by enhancing the function of antigen presenting cells and infiltration of activated CD8+ T cells and cytotoxic NK cells in TME 20, 23. Emerging evidence has showed that cancer cells evolve multiple mechanisms to destroy this pathway in order to evade immune surveillance 22, 24-26. So, the cGAS-STING pathway provides a new opportunity for pharmacological intervention against cancers.

In this study, we establish a novel indicator, immune-related prognostic index (IRPI), to predict the efficacy of HER2-targeted therapy and prognosis of HER2+ BC. We also explored new strategies to harness the immune system to promote anti-tumor effect in HER2+ BC with Herceptin-resistance.

## Materials and Methods

### Data collection

Data were extracted from the Gene Expression Omnibus (GEO) (http://www.ncbi.nlm.nih.gov/geo/) and The Cancer Genome Atlas (TCGA) (https://portal.gdc.cancer.gov/repository) databases. RNA expression of BC and corresponding clinical data were obtained from two GEO cohorts (GSE76360 and GSE191230) and TCGA cohort for subsequent analysis. GSE76360 contained 50 HER2+ BC patients who were enrolled on the 03-311 clinical trial. And clinical assessment included pathologic Complete Response (pCR), objective response (OBJR) and no response (NOR). GSE191230 contained 13 pretreated primary breast tumors and 7 distant metastasis breast tumors after Herceptin treatment. The data of 1068 BC patients from TCGA database, including 85 HER2+ BC patients was collated for further analysis. The single-cell RNA sequencing (scRNA-seq) data were obtained from GSE176078 cohort.

### Cell lines

MDA-MB-453 and SKBR3 cell lines were obtained from the American Type Culture Collection. Cells were cultured in complete Roswell Park Memorial Institute (RPMI) 1640 medium with 10% Fetal Bovine Serum (FBS) at 37 ℃ under 5% CO_2_.

### Co-expression network construction and modules associated with clinical traits identification

To discover the relationship between immune-related signaling and prognosis of HER2+ BC, the Weighted Gene Co-expression Network Analysis (WGCNA) for all genes was performed in HER2+ BC patients with one dose Herceptin treatment by using the “WGCNA” R package (version: 1.72). An adjacency matrix was clustered and co-expression modules were determined. The strongest positive correlation was selected for further analysis by calculating the Pearson correlation coefficient between the modules and clinical response.

### Gene set enrichment analysis (GSEA)

For gene set enrichment, the h.all.v7.4.symbols gene set was downloaded from the MSigDB database for enrichment analysis. Gene Ontology (GO) and HALLMARK analyses were conducted using “ClusterProfiler” R package. The output was visualized using “ggplot2” R package. The p value < 0.05 was considered statistically significant.

### Construction of the immune-related prognostic index (IRPI)

For constructing the IRPI, the “Limma” R package was used to identify the differentially expressed genes (DEGs) between parental and resistant MDA-MB-453 cell line with a false discovery rate (FDR) < 0.05 and |log2FC| ≥ 1 and DEGs before and after one dose of Herceptin treatment in the GSE76360 cohort with the FDR < 0.1. The intersection of DEGs were made to obtain the candidate genes. Univariate Cox regression analysis of OS was performed to identify prognostic genes with p <0.05 using TCGA HER2+ BC cohort. XGBoost algorithm was performed to identify immune-related genes. Finally, the Lasso Cox regression method was used to shrink the candidates to construct the most suitable signature with “glmnet” R package. The computational formula was as follows: IRPI_score = sum (each gene's expression * corresponding coefficient). The HER2+ BC patients were divided into high IRPI group and low IRPI group according to the median value of IRPI. The time-dependent ROC curve analysis was performed with “survivalROC” R package. The OS and PFS between different groups were compared by Kaplan-Meier analysis with “survival” and “survminer” R packages. Multivariate Cox regression analysis was also performed to investigate whether IRPI was an independent prognostic factor of OS. Other cohorts were used to validate the correlation between anti-HER2 therapy and IRPI.

### Tumor microenvironmental landscape analysis

To evaluate the TME, the proportion of different tumor-infiltrating cells were evaluated by xCell algorithm based on “immunedeconv” R package. The tracking tumor immunophenotype (TIP) analysis was performed on the TIP website (biocc.hrbmu.edu.cn/TIP/index.jsp) to evaluate immune activity during the “cancer-immunity cycle”. Correlations between IRPI and expression levels of several immune checkpoint molecules (ICMs) were also evaluated.

### scRNA-seq data analysis

To explore the relationship between IRPI and anti-tumor immunity, the scRNA-seq data of 4 primary HER2+ BC in GSE176078 cohort was analyzed with “Seurat” R package. After cell quality control and gene expression normalization with default parameters, each cell was annotated by canonical lineage markers, and cell clusters were displayed by Uniform manifold approximation and projection (UMAP) algorithms. Single-cell copy number variant (CNV) was evaluated with “inferCNV” R package and clusters with higher CNVs in epithelial cells were defined as tumor cells.

### Real-time quantitative PCR (qPCR)

Total RNA was isolated using EZ-press RNA Purification Kit. RNA quantity and quality were confirmed with a NanoDrop ND-1000 spectrophotometer, and cDNA was synthesized using the HiScript Ⅱ QRT SuperMix Kit. qPCR was performed according to the manufacturer's protocol (ChamQ SYBR qPCR Master Mix, Roche Apllied Science LightCycler 480). The relative amount of target gene mRNA was normalized to GAPDH. All qPCR reactions were done in quadruplicate. Gene-specific primers sequences listed in Table S1.

### Immunofluorescence assay

After all the treatments, cells growing on coverslips were washed twice with phosphate buffer saline (PBS) and fixed in 4% (vol/vol) paraformaldehyde for 10 mins and then permeabilized with 0.1% triton X-100. After blocking in 4% BSA for 45 mins at room temperature, cells were incubated with corresponding primary antibody overnight at 4℃. Cells were washed twice with PBS and incubated with secondary antibody for 1 hour at room temperature. Secondary antibody washed and stained by DAPI and mount on glass slides with ProLong™ Diamond Antifade Mountant (Invitrogen, P36970). The p-TBK1 results of xenografts from mice were analyzed by HALO modules from Indica Labs company.

### Flow cytometry

To observe the expression level of MHC-I on the cell membrane, parental and resistant cells treated for 48 hours with ADU-S100 were harvested and single-cell suspensions were washed twice in PBS. Cell surface staining was done with indicated fluorescent-labeled antibody for 30 min at 4 degrees and washed with PBS. All flow cytometry analysis was conducted on CytoFLEX (Beckman) and data analysis was performed using FlowJo (FlowJo Vx.0.7). All antibodies used for flow cytometry analysis were listed in Table S1.

### cGAMP measurement by ELISA

To measure the changes of intracellular cGAMP levels in parental and resistant cells after dsDNA treatment, cells were seeded on a six-well plate and after 18 hours stimulation with herring testis DNA (HT-DNA) (2 or 4μg/ml), cell lysates were prepared using Mammalian Protein Extraction Reagent (M-PER). The prepared samples were analyzed using Cayman 2'3'-cGAMP ELISA Kit. Quantification of 2'3'-cGAMP concentration was performed according to the manufacturer protocol. The reading was taken at 450nm by iMark Microplate Reader from BIO-RAD and calculated using a cGAMP standard curve.

### Immunoblotting

For western blotting, cells were harvested and lysed using RIPA buffer supplemented with phosphatase inhibitors. After sonicated, cell lysates were centrifuged at 12000g for 15 mins at 4 ℃ to remove insoluble material. A volume of 15-25 μg of total proteins was separated by 10% Bis-Tis polyacrylamide gels and transferred to PVDF membrane. The membrane was blocked with 5% milk and incubated with primary antibody overnight at 4 ℃. Antibodies for immunoblot were used at a dilution of 1:500-1:1000. Antibodies used in immunoblot were listed in Table S1.

### Co-culture experiment of peripheral blood mononucleated cells (PBMCs) with HER2+ BC cells

After blocking 24-well plates with CD3/CD28 antibodies overnight at 4 ℃, PBMCs including 10^6^ T cells were inoculated in AIM-V medium supplemented with Interleukin-2 (IL-2) and incubated for 3 hours. Then, 10^5^ cancer cells pretreated with or without STING agonist (STINGa) for 24 hours were added and co-cultured at 37 ℃ for 48 hours, adding DS-8201 or not. Washed gently with PBS twice and fixed with 4% paraformaldehyde for 15 mins, 0.005% crystal violet solution is used to visualize the living cancer cells. Data analysis was performed using ImageJ software.

### Human xenografts in NCG mice reconstituted with human lymphocytes

The 4-week-old female NCG (NOD/ShiltJGpt-Prkdc^em26Cd52^Il2rg^em26Cd22^/Gpt) mice were purchased from the Guangdong GemPharmatech Co., Ltd (Guangzhou, China). All animal experiments were conducted following the institutional guidelines and approved by our cancer center's Animal Care and Use Committee. NCG mice were injected subcutaneously with 5×10^6^ Herceptin-resistant SKBR3 cells. Once tumors had reached the target volume (30mm^3^), all mice were injected with 1×10^7^ human allogeneic PBMCs (day 0) intravenously from Milestone Biotechnologies company (Cat# P123020911C). Then mice were randomly assigned to treatment and control groups. On Day 2, mice received a single intraperitoneal injection of PBS (100 μl; as control) or DS-8201 (2mg/kg in 100 μl PBS). ADU-S100 (5 μg; 50 μl) was injected intratumorally every three days from Day 2 until three times. Tumors were measured at regular intervals with electronic calipers. Volumes were calculated by the formula: 0.5 × length × width × width in millimeters. After mice were sacrificed, the tumor tissues were excised and weighed. Jin (Zhengjun)'s formula was used to determine whether the combined effect of DS-8201 and ADU-S100 has a synergistic inhibitory effect on tumor growth. The formula is: Q = E_A+B_/(E_A_+E_B_-E_A_*E_B_) (Note: E_A_ is the effect of drug A, E_B_ is the effect of drug B, E_A+B_ is the combined effect) 27. Q value > 1.15 was synergistic; 0.85-1.15 was additive; < 0.85 was antagonistic.

### Immunohistochemical (IHC) staining

For human HER2+ BC analysis, 126 paraffin blocks of human HER2+ BC lesions with available follow-up data were selected for this study. All these samples were histopathologically diagnosed at the Sun Yat-sen University Cancer Center. All samples used in this study were approved by the medical ethics committee of Sun Yat-sen University Cancer Center. The Clinical characteristics of these patients were shown in Table S2. For SKBR3 tumor xenografts, the tumors tissues isolated from mice were fixed in 4% (vol/vol) paraformaldehyde overnight and embedded into paraffin block. The sections were dewaxed and rehydrated through graded alcohol to water before antigen unmasking, following by 3% hydrogen peroxide treatment. Then the slides were incubated with the diluted antibodies targeting human CD8, granzyme B (GZMB) and human IRF3 overnight at 4 ℃. After being washed, the sections were incubated with secondary antibodies for one hour at room temperature. Nuclei were counterstained with hematoxylin. For evaluating TILs in SKBR3 xenografts, images were captured in Nikon camera and software. The numbers of CD8+ TILs and GZMB+ TILs were measured in a random tumor area at 400-fold. The total number of tumor cells in the same field of view was calculated. The percents of CD8+ TILs and GZMB+ TILs were analyzed. The IHC staining results of xenografts were reviewed independently by two pathologists blinded to the treatment groups. To observe the nuclear translocation of IRF3 in human HER2+ BC, images were captured by Digital Pathology Slide Scanner (KF-PRO-020) from KFBIO company. The IHC staining results of human HER2+ BC were analyzed by HALO modules from Indica Labs company. The HER2+ BC patients were divided into high nuclear-IRF3 group (moderate and strong) and low nuclear-IRF3 group (negative and weak) according to the median value of H-score.

### Statistical analysis

All statistical analyses were conducted via R software (Version 4.2.2) or GraphPad Prism 9 software. Wilcox-test or student's t-test was used to analyze differences between two groups. Survival curves were compared with the log-rank test and described by Kaplan-Meier plots. Statistical significance was defined as p-value or adjust.p-value < 0.05, and all values were two-tailed.

## Results

### Down-regulation of type I IFN signaling is associated with poor prognosis in HER2+ BC patients

**Figure 1:**
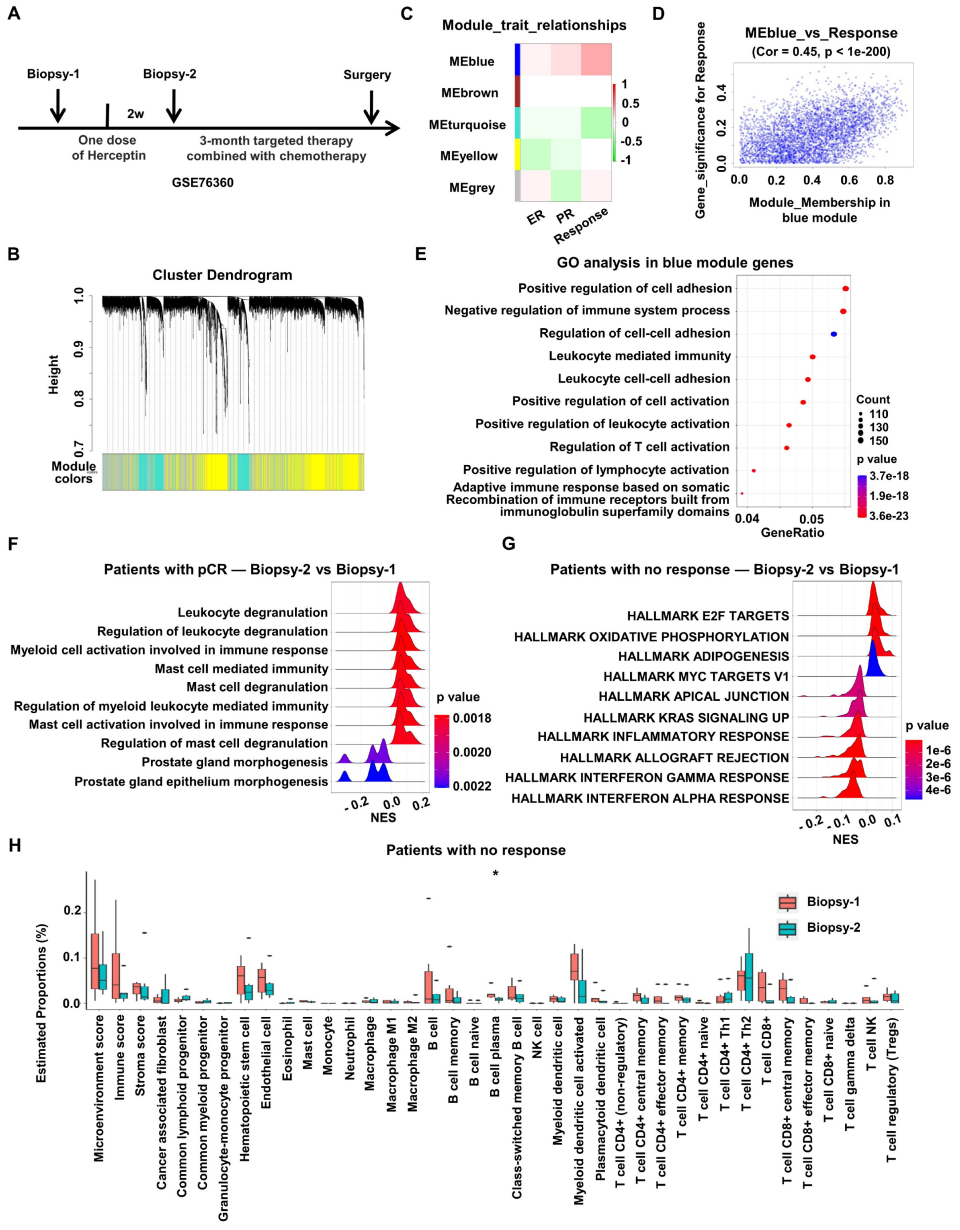
** Down-regulation of type I IFN signaling is associated with poor prognosis in HER2+ BC patients.** WGCNA analysis was performed using RNA expression data of BC treated with one dose of Herceptin in the GSE76360 cohort.** (A)** Schematic depiction of the GSE76360 cohort we used. **(B)** Gene dendrogram and modules before merging. **(C)** Pearson correlation analysis of merged modules and clinical characteristics. **(D)** Scatterplot of blue module and clinical response. **(E)** The bubble plot showed the enriched GO pathways of genes in blue module. **(F-G)** The ridge plot showed the gene set enrichment of DEGs before and after Herceptin treatment in BC patients with pCR **(F)** or no response **(G)**. **(H)** The boxplots showed the tumor microenvironmental components estimated by xCell algorithm in BC patients with no response. *p < 0.05. WGCNA: Weighted Gene Co-expression Network Analysis, BC: breast cancer, GO: Gene Ontology, DEGs: differentially expressed genes, pCR: pathologic complete response.

To explore how HER2-targeted therapy changes the characteristics of HER2+ BC, RNA expression and corresponding clinical data of HER2+ BC patients enrolled on the 03-311 trial were obtained from GSE76360 cohort. RNA expression profiling was conducted on tumor biopsies at baseline (Biopsy-1) and after brief-exposure to single-agent Herceptin (Biopsy-2), which could rule out other treatment interference (Figure 1A).

Firstly, to discover the gene modules most associated with the prognosis of HER2+ BC, WGCNA was performed using the expression profiles of total HER2+ BC patients after one dose of Herceptin treatment in the GSE76360 cohort (Biopsy 2) (Figure 1A). The soft threshold power value was set to 6 for subsequent analyses (Figure S1A). Dynamic module identification was performed and 5 co-expression modules were clustered, with the blue module having the strongest positive correlation with clinical response (Cor = 0.45, p < 1e-200) (Figure 1B-D, Figure S1B). The potential functions of total genes in the blue module were examined by GO enrichment. The results consisted of regulation of immune system process, including leukocyte mediated immunity, leukocyte cell-cell adhesion and regulation of immune cells activation. In addition, WGCNA analysis was also performed using the pre-treated data from GSE76360 cohort. Dynamic module identification was performed and 4 co-expression modules were clustered (Figure S1D), with the blue module having the strongest positive correlation with clinical response (Figure S1E). The potential functions of total genes in the blue module were examined by GO enrichment. The results consisted of several immune-related pathways, including Fc receptor signaling pathway (p value = 0.0057), myeloid leukocyte migration (p value = 0.0139) and macrophage derived foam cell differentiation (p value = 0.0141). The above results suggests that immune-related signaling was positively correlated with the prognosis of HER2+ BC (Figure 1E, Figure S1D-E).

Then we investigated the effect of anti-HER2 therapy on the immune microenvironment of HER2+ BC. DEGs before (Biopsy-1) and after one dose of Herceptin treatment (Biopsy-2) were identified to perform GSEA analysis in the GSE76360 cohort according to treatment response. The results showed that many immune-related biological processes, such as leukocyte degranulation, myeloid cell activation and mast cell mediated immunity, were obviously activated in patients with pCR after Herceptin treatment (Figure 1F), while multiple immune-related pathways, such as the IFN signaling, including IFN-alpha response pathway and IFN-gamma response pathway, were down-regulated in patients with no response (Figure 1G, Figure S1C). We also investigated changes of the tumor microenvironmental landscape after Herceptin treatment in patients with no response. The tumor microenvironmental components estimated by xCell algorithm indicated that tumor immune cell infiltration and scores showed a downward trend after targeted therapy in patients with no response, although not statistically significant (Figure 1H). In all, the immune-related signaling, especially the type I IFN signaling, would be down-regulated in HER2+ BC patients with poor prognosis after HER2-targeted therapy.

### Construction of an immune-related prognostic index (IRPI) for HER2+ BC

**Figure 2:**
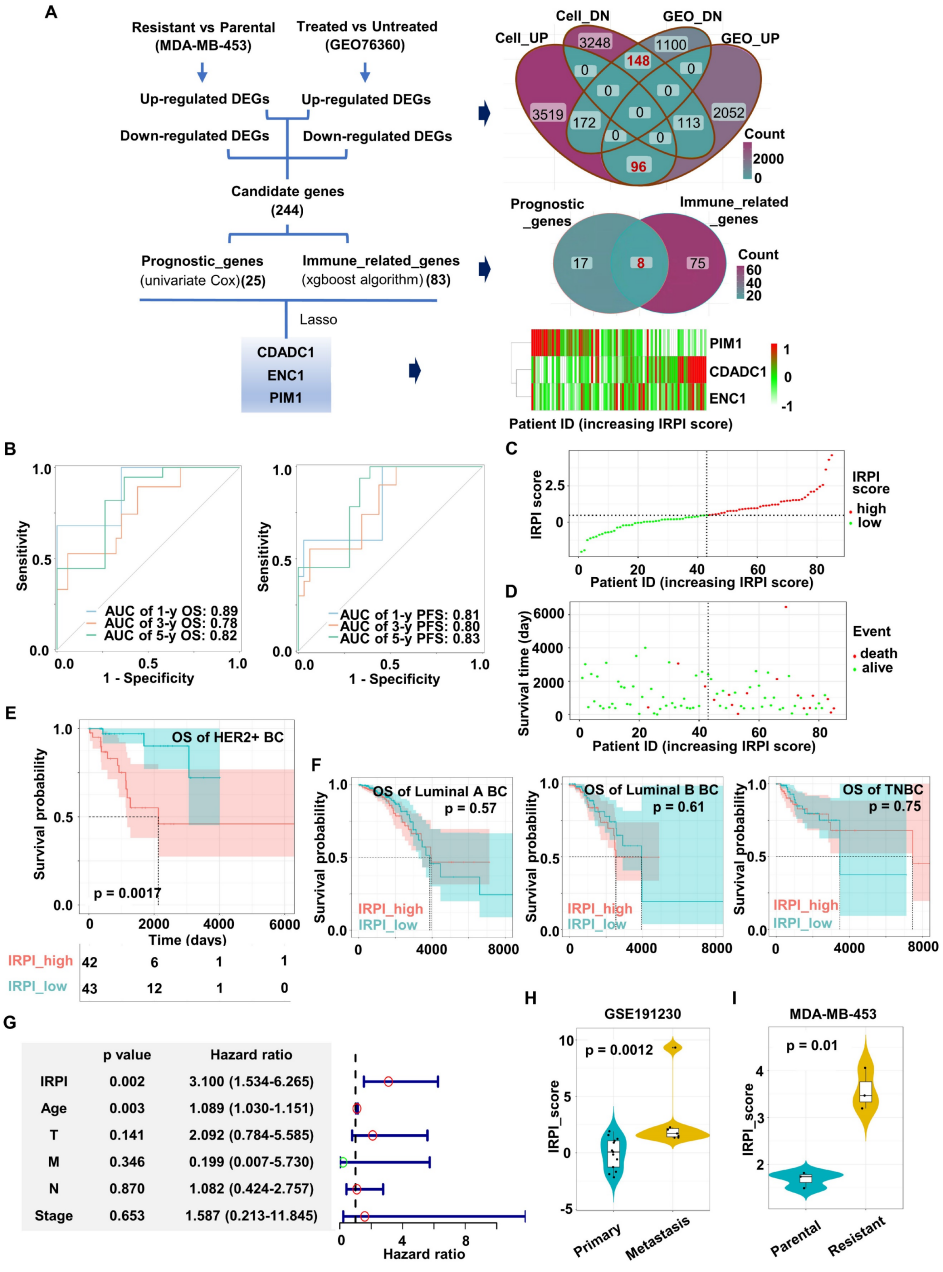
** Construction of an immune-related prognostic index (IRPI) for HER2+ BC. (A)** The flow chart for the construction of prognostic model (left). The Venn diagram showed the common genes of DEGs from cell line and GSE76360 cohort (top right). The Venn diagram showed the common genes of prognostic genes and immune-related genes (right center). The heatmap of the expression of three genes in IRPI according to IRPI score (bottom right). **(B)** AUC of time-dependent ROC curves at 1/3/5-year OS (left) and PFS (right) verified the prognostic accuracy of the IRPI in TCGA HER2+ BC cohort.** (C)** The HER2+ BC patients were divided into high and low IRPI group according to the median value of IRPI. **(D)** Distribution of IRPI score according to the survival status in TCGA HER2+ BC cohort. **(E-F)** The Kaplan-Meier estimate of the OS in different subtypes of BC, divided by two IRPI subtypes. **(G)** Results of the multivariate Cox regression analysis among the clinical characteristics and IRPI regarding OS in TCGA HER2+ BC cohort. **(H-I)** The boxplots showed the levels of IRPI score of patients in the validation data, including GSE191230 cohort **(H)** and MDA-MB-453 cell line **(I)**. Data was shown as mean ± SD. And p values are based on Wilcoxon test. BC: breast cancer, DEGs: differentially expressed genes, Cell_UP/ Cell_DN: up- or down-regulated DEGs between Herceptin-resistant and parental MDA-MB-453, GEO_UP/ GEO_DN: up- or down-regulated DEGs between Herceptin-treated and untreated BC in the GSE76360 cohort, OS: overall survival, PFS: progression-free survival, TCGA: The Cancer Genome Atlas.

To fully consider immune infiltration of tumor and better select HER2+ BC patients probably benefit from Herceptin treatment, an IRPI was constructed for further stratification. The flow diagram of index construction is showed in the Figure 2A. Firstly, Herceptin-resistant HER2+ BC cell lines were established by low dose Herceptin treatment (5 μg/ml) for more than 6 months (Figure S2A; Figure S3A).

Colony formation assays showed that the colony formation ability of parental MDA-MB-453/SKBR3 cells was significantly inhibited with the increase of Herceptin in the medium, while the colony formation ability of resistant MDA-MB-453/SKBR3 cells was not reduced in the same condition (Figure S2B-C). RNA sequence of MDA-MB-453 cell line was performed, and DEGs between parental and resistant MDA-MB-453 cell line were identified. Secondly, the intersection of DEGs before and after Herceptin treatment in the GSE76360 cohort and DEGs from MDA-MB-453 cell line are displayed in the Venn diagram (Figure 2A). 244 candidate genes were obtained, including 96 up-regulated and 148 down-regulated DEGs (Figure 2A). Thirdly, TCGA cohort containing 85 HER2+ BC samples with OS information was used for prognostic model construction. 25 out of 244 candidate genes significantly correlated with OS using univariate Cox regression analysis. Bagaev *et al.* have identified four TME subtypes (MFP subtypes) that are conserved across diverse cancers and correlate with immunotherapy response 28. In our study, XGBoost algorithm was applied to identify 83 immune-related genes according to this MFP subtypes (Figure S2G). The intersection of these two outputs included 8 genes (Figure 2A). Finally, a 3-gene signature (CDADC1, ENC1, PIM1) was eventually constructed by Lasso Cox regression analysis using TCGA HER2+ BC cohort (Figure 2A, Figure S2D-F). The IRPI score of each patient was calculated with the formula below. IRPI_score = (0.297944181 * CDADC1 exp.) + (0.004639381 * ENC1 exp.) - (0.046579753 * PIM1 exp.). As shown in Figures 2A, CDADC1 and ENC1 were shown as risk factors, while PIM1 was shown as a protective factor.

Next, we validated the predictive power of our prognostic model in the TCGA HER2+ BC cohort. The time-dependent area under ROC curve (AUC) for OS reached 0.89 at 1 year, 0.78 at 3 years, and 0.82 at 5 years, while the time-dependent AUC for PFS reached 0.81 at 1 year, 0.80 at 3 years, and 0.83 at 5 years (Figure 2B). Furthermore, the HER2+ BC patients were divided into high IRPI group (n = 42) and low IRPI group (n = 43) by the median IRPI score as cut-off value (Figure 2C). Patients with high IRPI had a higher probability of death than patients with low IRPI (Figure 2D). Kaplan-Meier analysis also showed that high IRPI patients had a worse OS only in HER2+ BC but not in other BC subtypes (Figure 2E-F). More importantly, the IRPI was determined as an independent prognostic factor among the clinical characteristics by multivariate Cox regression (Figure 2G). We further assessed the association between IRPI and Herceptin-resistance in HER2+ BC in external validation cohorts. The IRPI of distant metastasis of HER2+ BC was obviously higher than the IRPI of primary BC in GSE191230 cohort (Figure 2H). Moreover, the IRPI was significantly elevated in Herceptin-resistant MDA-MB-453 (Figure 2I). Together, an immune-related index (IRPI) was constructed to predict the prognosis of HER2+ BC effectively, which was highly related to Herceptin resistance.

### HER2+ BC patients with high IRPI have suppressive immune microenvironment landscape

**Figure 3:**
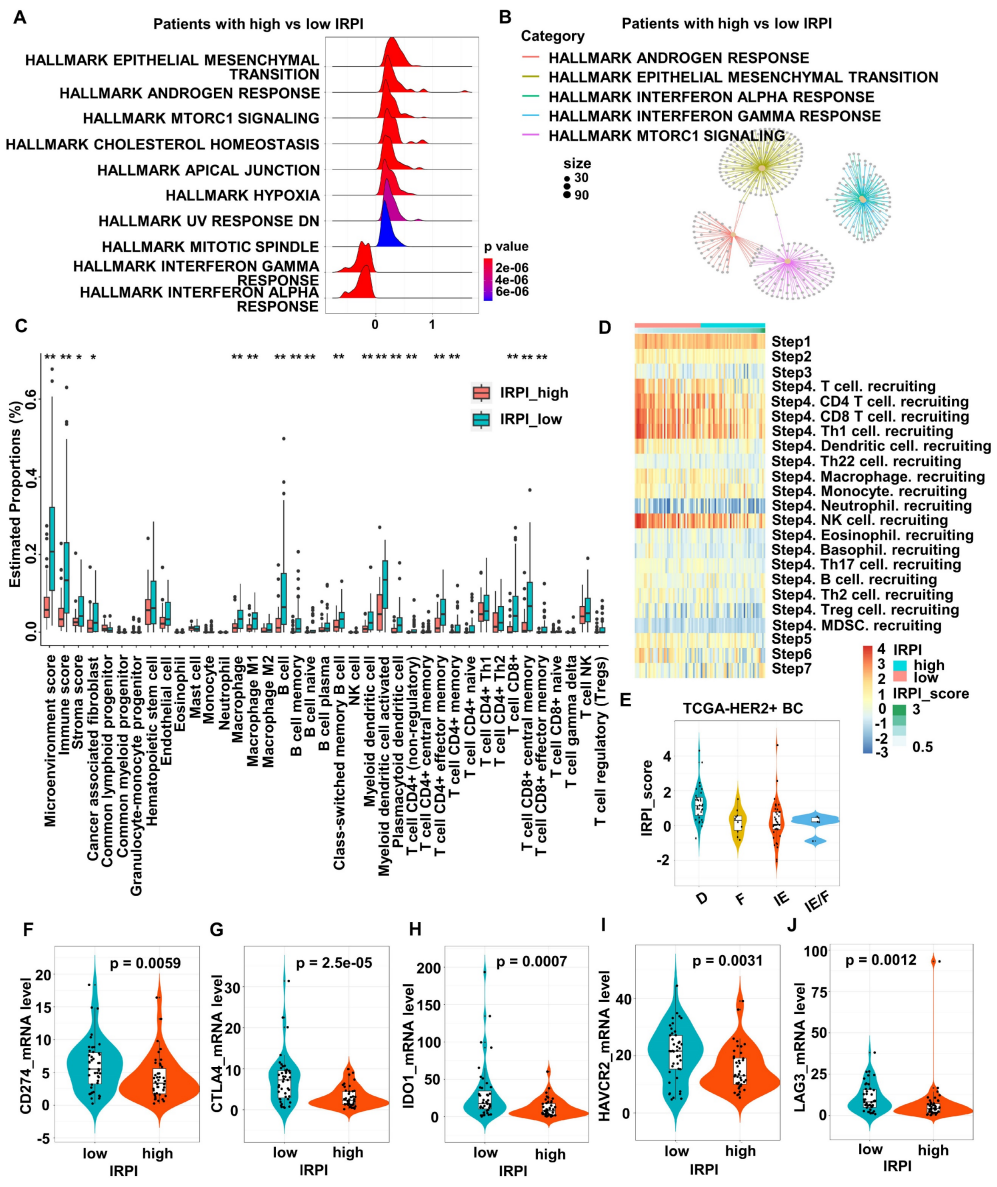
** HER2+ BC patients with high IRPI have suppressive immune microenvironment landscape. (A)** The ridge plot showed the HALLMARK enrichment of DEGs between patients with high IRPI and low IRPI in the TCGA HER2+ BC cohort. **(B)** The network plot showed the enriched functional modules of DEGs between patients with high IRPI and low IRPI. **(C)** The boxplot showed the tumor microenvironmental components estimated by xCell algorithm in high and low IRPI patients in the TCGA HER2+ BC cohort. **(D)** The heatmap showed the correlation between IRPI and the tracking tumor immunophenotype. **(E)** The boxplot showed the levels of IRPI score of patients with MFP immune subtypes. **(F-J)** The expression levels of immune checkpoint molecules in patients with high and low IRPI in the TCGA HER2+ BC cohort. Data was shown as mean ± SD. And p values are based on Wilcoxon test. **p < 0.01, *p < 0.05. DEGs: differentially expressed genes, TCGA: The Cancer Genome Atlas, BC: breast cancer.

To explore the relationship between IRPI and TME, DEGs between the high and low IRPI groups in TCGA HER2+ BC cohort were used to perform gene sets enrichment. The results showed that IFN signaling, especially type I IFN response pathway, was down-regulated in the high IRPI patients (Figure 3A). The functional modules enriched by these DEGs also included the IFN-alpha response pathway showed by network diagram (Figure 3B). Since preclinical and clinical research had confirmed the essential role of IFNs for effective host immunological responses to malignant cells, we further screened the TCGA HER2+ BC cohort by xCell algorithm to explore the relationship between IRPI and TME landscape. As shown in Figure 3C, patients with low IRPI had higher immune scores and stroma scores. In detail, patients with low IRPI had higher proportions of cancer associated fibroblasts, macrophages, M1 macrophages, B cells, memory B cells, naïve B cells, class-switched memory B cells, myeloid dendritic cells, activated myeloid dendritic cells, plasmacytoid dendritic cells, CD4+ T cells (non-regulatory), effector memory CD4+ T cells, memory CD4+ T cells, CD8+ T cells, central memory CD8+ T cells and effector memory CD8+ T cells (Figure 3C). Consistently, IRPI level was significantly associated with the infiltration of immune cells, especially CD8+ T cells, Th1 cells and NK cells during the cancer-immunity cycle (Figure 3D). Our results also indicated that the immune-enriched/non-fibrotic (IE) subtype had the lowest IRPI level compared to other MFP subtypes 28 (Figure 3E). Meanwhile, correlations between IRPI and several crucial ICMs were investigated. The expression levels of ICMs such as PD-L1, CTLA4, IDO1, HAVCR2 and LAG3 in low IRPI group were all higher than those in high IRPI group (Figure 3F-J). The above results confirm that HER2+ BC patients with high IRPI have suppressive tumor immune microenvironment.

### scRNA-seq analysis shows down-regulation of type I IFN response pathway in tumor cells with high IRPI

**Figure 4:**
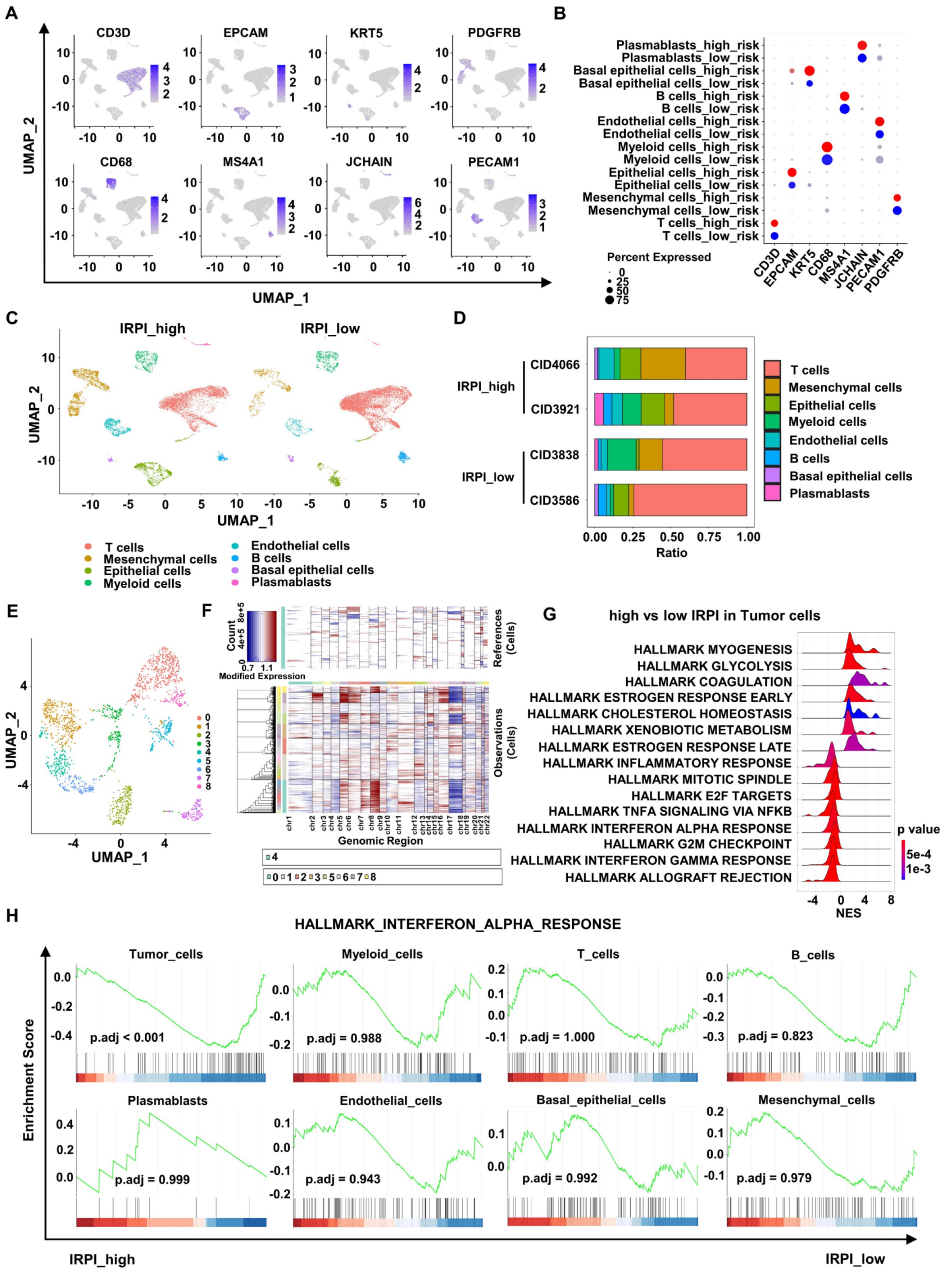
** scRNA-seq analysis shows down-regulation of type I IFN response pathway in tumor cells with high IRPI. (A)** Log-normalized expression of canonical markers for cells in 8 clusters in the GSE176078 cohort. **(B)** Bubble plot of the percent expression of marker genes in different cell subtypes. **(C)** UMAP visualization of all cell subtypes in HER2+ BC patients with high and low IRPI. **(D)** Bar plots of the cell proportions of each HER2+ BC patients. **(E)** UMAP visualization of reclustered epithelial cells in all HER2+ BC patients.** (F)** The heatmap showed that cells in cluster 0, 5, 7 and 8 of epithelial cells had higher CNVs, which were defined as tumor cells.** (G)** The ridge plot showed the HALLMARK enrichment of DEGs between tumor cells in high IRPI group and low IRPI group. **(H)** GSEA indicated that Tumor cells, but not other non-tumor cells, showed down-regulation of type I IFN response pathway in HER2+ BC with high IRPI. scRNA-seq: single-cell RNA sequencing, UMAP: Uniform manifold approximation and projection, CNVs: copy number variants, DEGs: differentially expressed genes, GSEA: Gene set enrichment analysis.

To further explore the relationship between IRPI and anti-tumor immunity landscape, we analyzed scRNA-seq data of 4 primary HER2+ BC in the GSE176078 cohort. These 4 patients were divided into two groups based on their corresponding bulk RNA sequence data, with CID4066 and CID3921 belonging to high IRPI group and CID3838 and CID3586 belonging to low IRPI group. After quality control analysis performed, a total of 16,803 single cells were annotated using canonical lineage markers (Figure 4A-B). UMAP visualization showed a higher mesenchymal cells infiltration in high IRPI group, while tumors in low IRPI group had a higher T cells infiltration (Figure 4C-D). To distinguish the tumor cells, UMAP visualization of epithelial cells were reclustered (Figure 4E). We estimated CNV profiles using “inferCNV” R package. The results showed that cluster 0, 5, 7 and 8 had higher CNVs and they were considered as tumor cells (Figure 4F). Next, enrichment analysis of DEGs between high and low IRPI in tumor cells showed that the allograft rejection pathway, G2M checkpoint pathway and most importantly, the type I IFN response pathway were all down-regulated in high IRPI tumor cells (Figure 4G). Moreover, the type I IFN response pathway were not down-regulated in other non-tumor cells of the high IRPI BC (Figure 4H).

### cGAS-STING signaling is inactive in Herceptin-resistant HER2+ BC

**Figure 5:**
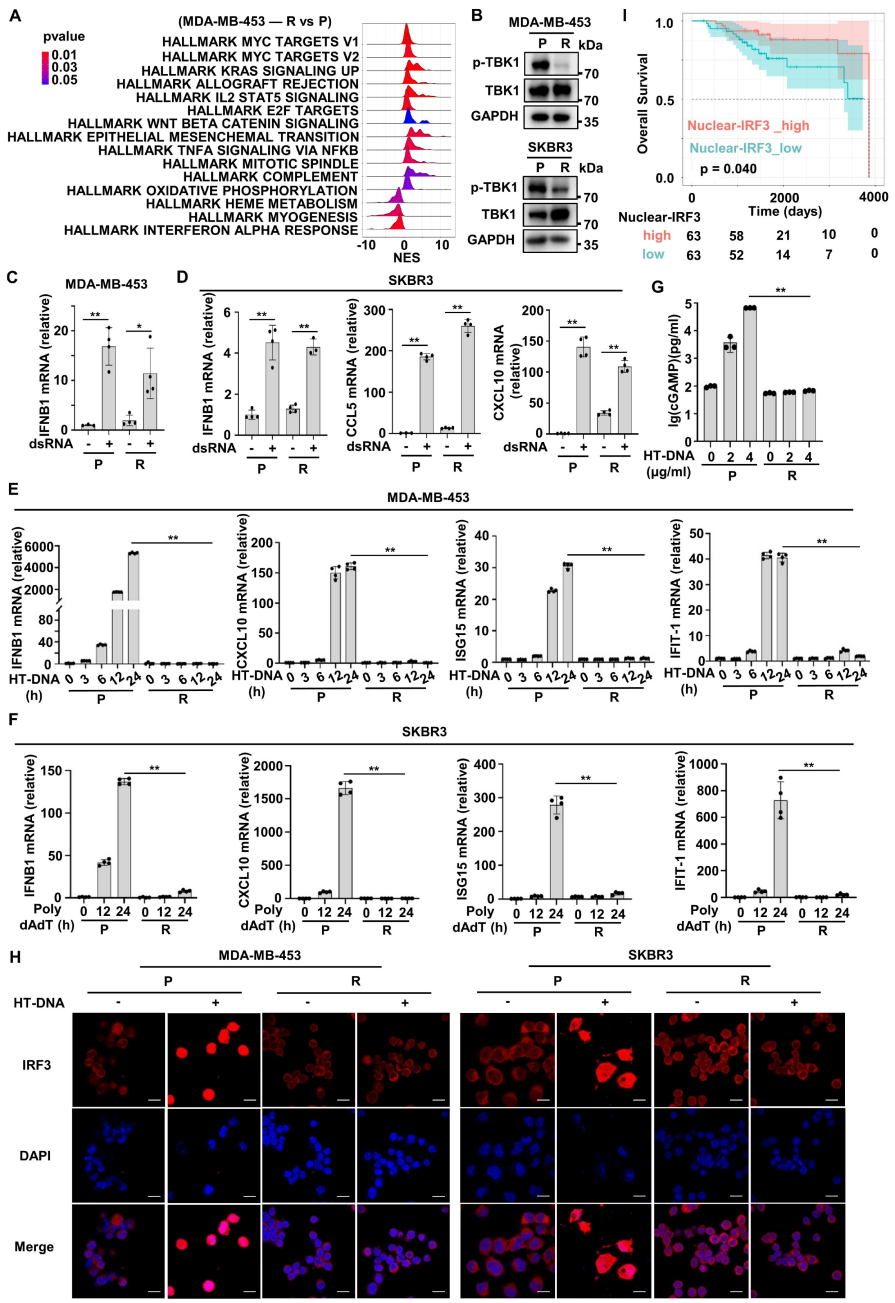
** cGAS-STING signaling is inactive in Herceptin-resistant HER2+ BC. (A)** The ridge plot showed the HALLMARK enrichment of DEGs between Herceptin-resistant and parental MDA-MB-453.** (B)** Western blot analysis of the activation of TBK1 in parental and Herceptin-resistant cell lines. These assays were repeated three times. **(C-D)** MDA-MB-453 **(C)** and SKBR3 **(D)** were treated with 200 ng/ml dsRNA for 24 hours and harvested for qPCR analysis.** (E-F)** qPCR analysis of IFN-β and downstream ISGs mRNA expression in parental and Herceptin-resistant cell lines transfected with 4 μg/ml HT-DNA** (E)** or 800 ng/ml Poly dAdT** (F)** for the times indicated.** (G)** Parental and resistant SKBR3 cells were treated with 4 μg/ml HT-DNA for 6 hours and harvested for ELISA detection of cytoplasmic cGAMP. **(H)** MDA-MB-453 and SKBR3 were transfected with 4 μg/ml HT-DNA for 12 hours, and the translocation of endogenous IRF3 was taken by confocal microscopy at the same magnification (63×/1.4 NA oil immersion objective). Cells were stained for IRF3 (red) and DNA is visualized with DAPI (blue). Scar bar: 20 μm. **(I)** Kaplan-Meier plots of the overall survival of HER2+ BC patients, stratified by the median value of IHC H-score of nuclear-IRF3 staining. Data was shown as mean ± SD. And p values are based on Student's t test. **p < 0.01, *p < 0.05. P: parental cells; R: resistant cells. DEGs: differentially expressed genes, dsRNA: double-strand RNA, qPCR: Quantitative real-time PCR, ISGs: IFN-stimulated genes.

The above bioinformatics analysis indicated that HER2+ BC patients with high IRPI had a down-regulated type I IFN response pathway in tumor cells and showed resistance to Herceptin. Next, we further confirmed this analysis using established Herceptin-resistant cell lines including MDA-MB-453 and SKBR3. Enrichment analysis showed that type I IFN response pathway, especially the IFN-alpha response pathway was down-regulated mostly in resistant MDA-MB-453 cell line compared with parental cell line (Figure 5A). The IFN-alpha response pathway plays a pivotal role in innate immunity system required for effective anti-tumor immunity 29. As expected, the activation of TBK1, the core upstream protein of IFN-alpha signaling, was inhibited in resistant strains (Figure 5B). Since RNA from dying cells and cytoplasmic DNA fragments may induce a robust activation of the endogenous IFN system in tumor 19, we next assessed whether the sensing pathways of these nucleic acids were altered in resistant cells. We transfected double-strand RNA (dsRNA) or herring testis DNA (HT-DNA) into parental and resistant cells. The results showed dsRNA transfection significantly increased the expression of IFNB1 mRNA and downstream ISGs mRNA in resistant cells, which was similar to that in parental cells (Figure 5C-D). In contrast, HT-DNA transfection could only increase the expression of IFNB1 mRNA and ISGs mRNA in parental cells, but not in resistant cells (Figure 5E-F). We further detected the intensity of micronuclei in cytoplasm of the parental and Herceptin-resistant cells. The results showed that resistant cell line had a higher intensity of micronuclei in cytoplasm (Figure S3C). Moreover, the expression of cGAS protein had no significant difference between parental and resistant cells (Figure S3B)**.** We then measured intracellular cGAMP levels by ELISA and found that after HT-DNA treatment, cGAMP could significantly increase in parental cells, but not resistant cells (Figure 5G). These results suggest that cGAS-STING pathway, the major innate immune pathway for sensing cytosolic DNA and activating IFN signaling, may be suppressed in resistant cells. As IRF3 is the key transcription factor of cGAS-STING pathway 30, we next examined IRF3's subcellular localization. Normally, IRF3 was mainly present in the cytoplasm. Actually, IRF3 was translocated into the nucleus after HT-DNA exposure in the parental cells (Figure 5H). In contrast, IRF3 translocation to the nucleus was significantly reduced in the resistant cells when treated with HT-DNA (Figure 5H, Figure S3D). To assess the clinical significance of cGAS-STING signaling in HER2+ BC samples, 126 paraffin blocks of human HER2+ BC lesions with available follow-up data were selected for this study (Table S2). The protein levels of nuclear IRF3 were evaluated by IHC (Figure S3E). The Kaplan-Meier survival analysis showed that patients with high expression of nuclear IRF3 had longer OS than those in the low-expression group (Figure 5I). Overall, these results illustrate that cGAS-STING signaling is suppressed in Herceptin-resistant HER2+ BC.

### Activation of IFN signaling by STING agonists reverses cGAS-STING pathway activity in Herceptin-resistant HER2+ BC

**Figure 6:**
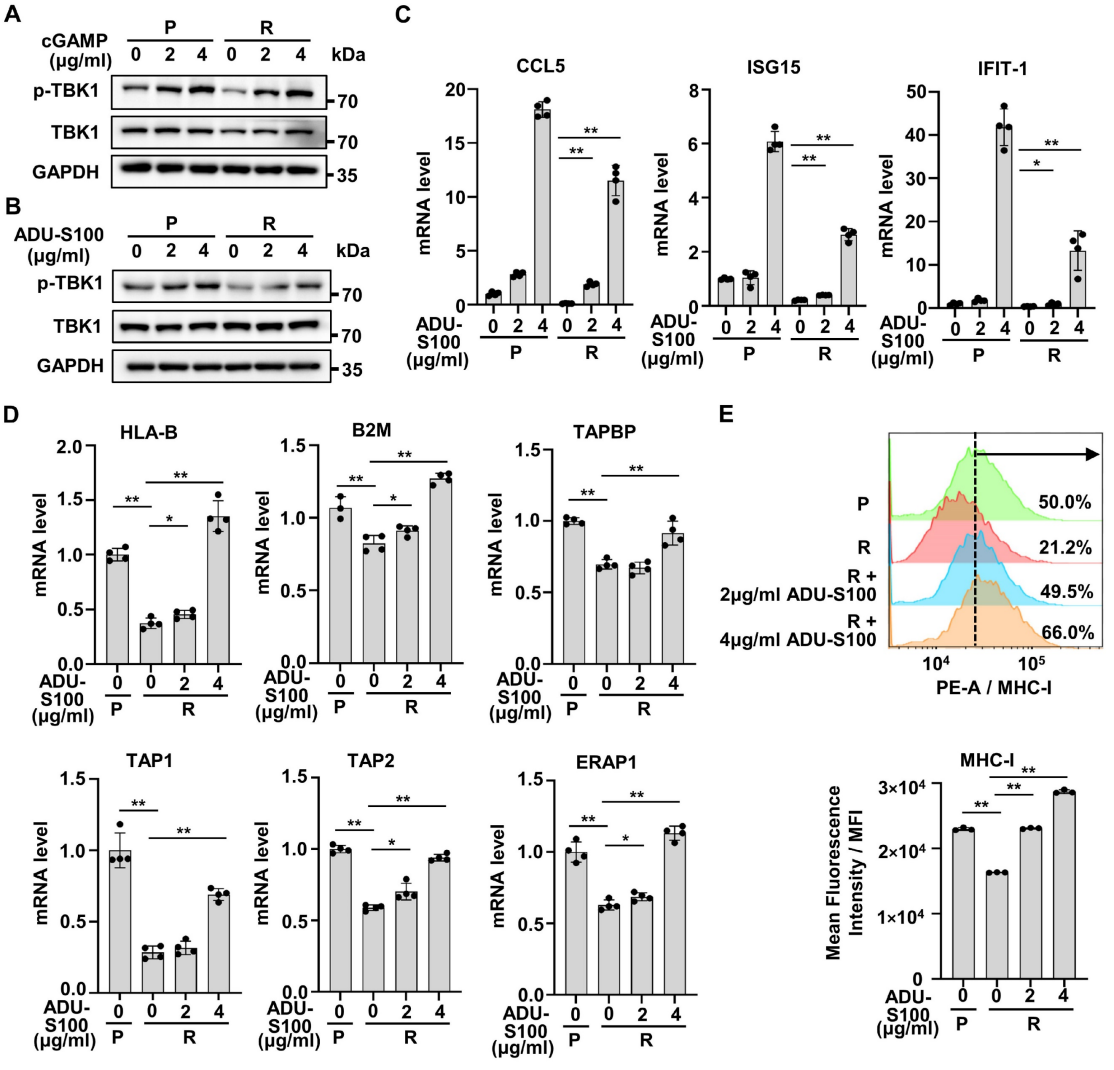
** Activation of IFN signaling by STING agonists reverses cGAS-STING pathway activity in Herceptin-resistant HER2+ BC. (A-B)** Parental and resistant SKBR3 cells were treated with cGAMP for 6 hours **(A)** or ADU-S100 for 24 hours **(B)**, and harvested for western blot analysis. These assays were repeated three times. **(C)** Parental and resistant SKBR3 were treated with ADU-S100 for 24 hours, and harvested for qPCR analysis of downstream ISGs mRNA expression. **(D)** Parental and resistant SKBR3 cells were treated with ADU-S100 for 24 hours, and harvested for qPCR analysis of mRNA expression of genes involved in antigen presentation. **(E)** Flow cytometry measuring MHC-I protein on the cell membrane with ADU-S100 at different concentrations for 24 hours using SKBR3 cell line. Data was shown as mean ± SD. And p values are based on Student's t test. **p < 0.01, *p < 0.05. P: parental cells; R: resistant cells, qPCR: Quantitative real-time PCR, ISGs: IFN-stimulated genes.

Activation of cGAS-STING pathway promotes the transcription of type I IFNs and has previously been reported to promote anti-tumor immunity in many preclinical models 31-33. As the production of cGAMP was down-regulated with HT-DNA treatment in resistant cells, we further treated resistant cells with cGAMP and found that cGAMP could significantly increase the phosphorylation of TBK1 in resistant cells, just like in parental cells (Figure 6A).

A variety of STINGa have been approved for clinical trials 34, 35. ADU-S100, one of the STINGa, is a synthetic cyclic dinucleotide that activates the cGAS-STING pathway 35. We tried to examine whether ADU-S100 could reactivate IFN signaling in resistant cells. The results showed that ADU-S100 could significantly increase the phosphorylation of TBK1 in both parental and resistant cells (Figure 6B). Also, ADU-S100 could partially reactivate the mRNA expression of ISGs in resistant cells (Figure 6C). Since activation of IFN signaling is essential for antigen presentation and anti-tumor immunity 19, we analyzed changes of antigen presenting ability in resistant cells after STINGa treatment. Compared with parental cells, the mRNA levels of genes involved in antigen presentation were significantly reduced in resistant cells, but could be restored by ADU-S100 intervention (Figure 6D). MHC-I expressed on the cell surface is one of the key tumor antigen presentation machinery components and is indispensable for T cell recognition and killing 19, 36. Consistent with results in mRNA level, we detected a low level of MHC-I in resistant cells compared with parental cells, as well as increased levels of MHC-I in resistant cells after ADU-S100 treatment by flow cytometric assay (Figure 6E). Taken together, these results suggest that STINGa could induce robust reactivation of cGAS-STING pathway in Herceptin-resistant HER2+ BC.

### The combination of STINGa and DS-8201 enhances immune surveillance to inhibit tumor growth of Herceptin-resistant HER2+ BC

**Figure 7:**
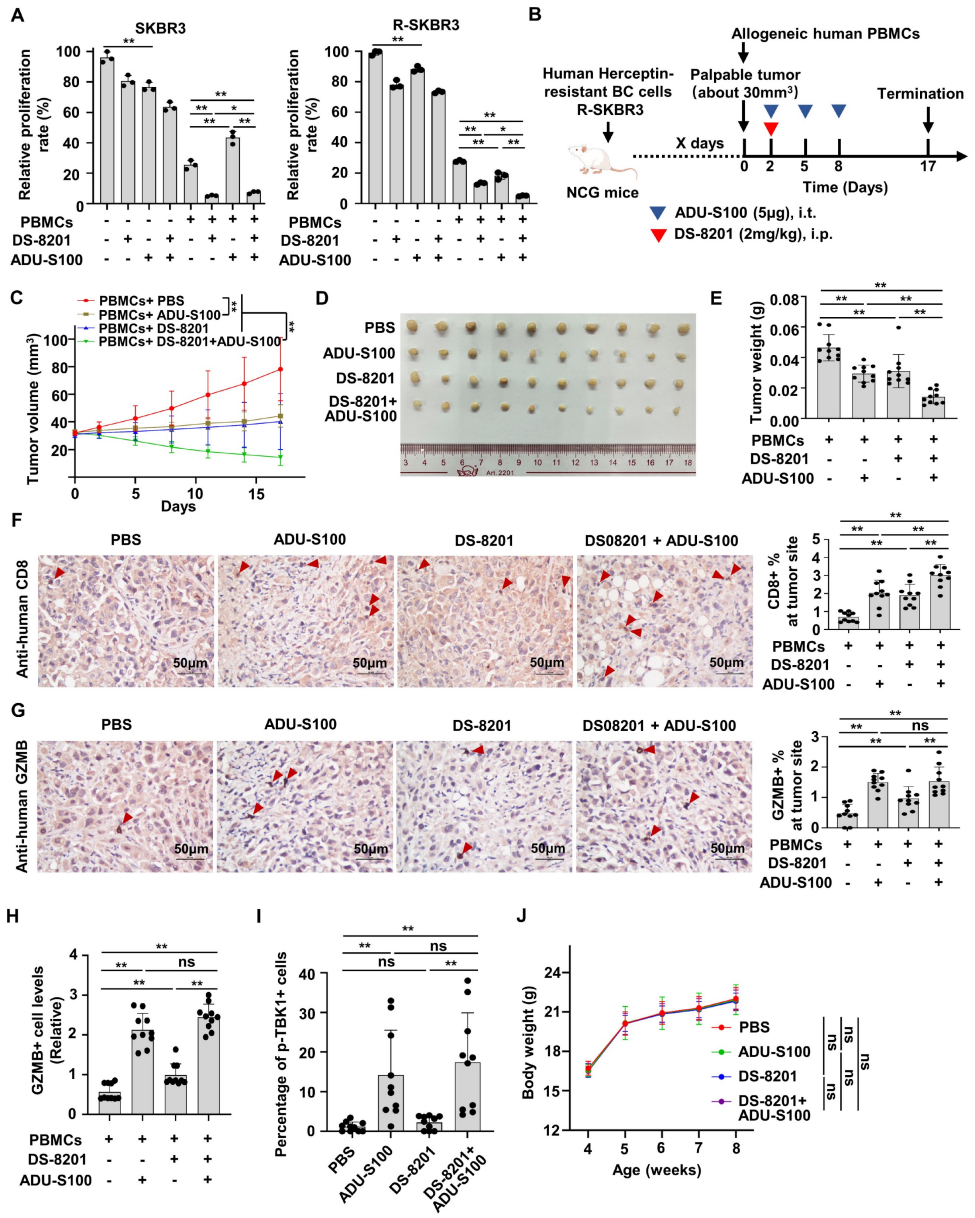
** The combination of STINGa and DS-8201 inhibits tumor growth of Herceptin-resistant HER2+ BC. (A)** Co-culture experiments of PBMCs from healthy human donors with parental or Herceptin-resistant cells treating with combination of ADU-S100 (4μg/ml) and DS-8201 (10μg/ml).** (B-E)** Tumor growth of Herceptin-resistant SKBR3 cells in humanized NCG mice following treatment with ADU-S100 and/or DS-8201. The *in vivo* experimental layout** (B)**. Tumor volumes were calculated **(C)**, and tumor weights from experiment on autopsy **(D-E)**.** (F-G)** Representative images of IHC staining of CD8+ lymphocytes** (F)** and GZMB+ lymphocytes** (G)** in Herceptin-resistant SKBR3 xenograft tumor sections after treatment (left, n = 10 mice per groups). Quantitative IHC analysis of percent of CD8+ lymphocytes and GZMB+ lymphocytes at tumor site (right). **(H)** Expression of GZMB in xenografts from mice was detected by immunofluorescence assay. **(I)** Expression of p-TBK1 in xenografts from mice was detected by immunofluorescence assay. **(J)** The weight of mice during the experiment in different groups. Data was shown as mean ± SD. And p values are based on Student's t test. **p < 0.01, *p < 0.05. ns: non-significant, i.t.: Intratumoral injection, i.p.: intraperitoneal injection, PBMCs: peripheral blood mononucleated cells, IHC: Immunohistochemical, BC: breast cancer, GZMB: granzyme B.

Increasing evidence supports that DS-8201 is the standard of care for HER2+ BC patients with Herceptin-resistance 3. We proved that STING signaling is suppressive in Herceptin-resistant HER2+ BC and that STINGa could release the activation of type I IFN response pathway. Therefore, we speculated that the combination of DS-8201 and STINGa might have a synergistic anti-tumor effect in the presence of PBMCs. We firstly investigate the effect of ADU-S100 alone in the parental and resistant cells. Interestingly, although ADU-S100 increased cell death in both parental and resistant cells, ADU-S100 alone increased more cell death in parental cells than that in resistant cells. But co-culture experiment with PBMCs showed that ADU-S100 alone promoted the death of cancer cells only in the Herceptin-resistant SKBR3 cells, but not in the parental cells (Figure 7A). We further examined whether STINGa has a synergistic anti-tumor effect with DS-8201. As expected, co-culture experiment with PBMCs demonstrated that DS-8201 and ADU-S100 had a significantly increased anti-tumor efficacy compared with DS-8201 or ADU-S100 alone in Herceptin-resistant cells, but not in parental cells (Figure 7A). To validate the combined effect of DS-8201 and STINGa *in vivo*, we tested them in immunodeficient NCG mice bearing Herceptin-resistant tumors and reconstituted with human allogeneic PBMCs. Herceptin-resistant SKBR3 cells were injected into NCG mice subcutaneously. Once tumor xenografts were established (about 30mm^3^), human PBMCs were injected intravenously (day 0) and mice were treated with DS-8201 and ADU-S100 as indicated (Figure 7B). Consistent with the observation *in vitro*, treatment with either DS-8201 or ADU-S100 had a minimal effect, but combination of DS-8201 and ADU-S100 significantly improved tumor growth inhibition with synergistic anti-tumor effects (q = 1.21), as confirmed by the growth curves of xenograft tumor volumes and tumor weights (Figure 7C-E). We investigated the influence of this combined treatment strategy on anti-tumor immunity. Indeed, the combined treatment led to increased CD8+ TIL populations (Figure 7F). The combined treatment also led to an increased GZMB release compared to single DS-8201 treatment (Figure 7G). To further confirm the phenomena, Immunofluorescence assay was also performed to determine GZMB release. And the results were similar to those by IHC assay (Figure 7H, Figure S4B). Furthermore, to analyze the relationship between cGAS-STING signaling expression and the cytotoxicity of DS-8201, the levels of phosphorylated TBK1 in xenograft tumor sections from Figure 7B were detected by immunofluorescence assay. The results showed that compared with control group and DS-8201 treatment group, phosphorylation of TBK1 in EpCAM+ xenograft tumor cells was much higher in the ADU-S100 treatment group and combined treatment group (Figure 7I, Figure S4C). No abnormalities in the general condition were observed in mice during the experiment. The weight of mice in different groups was measured. And the results showed no significant difference in body weight between treatment groups and control group (Figure 7J), indicating the combination treatment has no obvious toxic effect on mice. Together, these data indicate that STINGa has the potential to enhance the efficacy of DS-8201 in Herceptin-resistant HER2+ tumors *in vivo*.

## Discussion

In this study, we construct an immune-related prognostic index (IRPI) to select HER2+ BC patients probably benefit from Herceptin treatment. BC patients with high IRPI have a suppressive tumor immune microenvironment and show resistance to Herceptin. Further bioinformatics analysis and experimental verification show that silencing of the cGAS-STING pathway is a key determinant of immune escape in Herceptin-resistant BC. Importantly, activation of cGAS-STING pathway by STINGa in Herceptin-resistant BC could reverse IFN signaling activity, which promotes anti-tumor immune response and have a synergistic anti-tumor effect with DS-8201 *in vitro* and *in vivo*. To the best of our knowledge, this study is the first to propose the strategy of combining STINGa and DS-8201 in HER2+ BC with Herceptin resistance.

Given the heterogeneity of Herceptin-treatment response, identifying biomarkers of response beyond HER2 status is highly needed. Indeed, efforts to establish validated biomarkers of response to anti-HER2 therapy based on transcriptome analysis have met with limit success in other studies 37-39. Meanwhile, anti-HER2 therapy could induce local and systemic immunomodulation, which is associated with clinical outcomes 10, 40. In this study, we reasoned that the impact of immune infiltration on prognosis was not fully considered. Thus, we considered the addition of these two factors, evaluation of immune infiltration and regulation of TME by Herceptin-treatment, would improve our ability to uncover features associated with response to anti-HER2 therapy. So, we created an immune-related 3-gene (CDADC1, ENC1, PIM1) index (IRPI), an independent prognostic factor among multiple clinical characteristics, and found that it could predict OS and PFS of HER2+BC patients. CDADC1 encodes a subunit of cytidine and dCMP deaminase, which involved in DNA cytosine deamination and cytidine deamination 41. ENC1 overexpression was found to be associated with high metastasis and poor prognosis in BC patients by modulating β-catenin pathway 42. PIM1 is a serine/threonine kinase and able to promote tumor growth and drug resistance 43. Targeting PIM1 could down-regulate the expression of HER2 and circumventing lapatinib resistance in HER2+ BC 43. Consistent with previous finding 41, 42, our study found that the relatively higher expression of ENC1 and CDADC1 was associated with a poor prognosis. Meanwhile, the IRPI was significantly elevated in Herceptin-resistance BC, indicating the close relationship between the IRPI and anti-HER2 resistance. Furthermore, the HER2+ BC in low IRPI group tended to be immunologically “hot”, which was more likely to benefit from anti-tumor immunity, while Herceptin-resistant BC with high IRPI tended to be immunologically “cold”, which was hard to benefit from anti-tumor immunity 44. To a certain extent, our discovery explains the poor efficacy of immunotherapy in the clinical trials for Herceptin-resistant HER2+ BC 7, 45. Consistent with a favorable benefit of immunotherapy in HER2+ BC patients with PD-L1-positive tumors in clinical trials 45, 46, HER2+ BC in low IRPI group tended to have high expression level of ICMs such as PD-L1, CTLA4, IDO1, HAVCR2 and LAG3, illustrating the feasibility and power of our index.

In our study, the innate immune pathways, especial type I IFN pathway, were down-regulated in Herceptin-resistant BC with high IRPI. Plenty of evidence indicated that the type I IFN signaling plays a crucial role in antigen presentation and effective host immunological responses in cancer 18. However, cancer cells tend to interfere with this signaling for evasion of immune surveillance 22. Ong L.T. *et al.* demonstrated an epigenetic regulatory mechanism suppressing the expression of the IFI16-CXCL10/11 signaling and activation of IFN response that making HER2+ BC resistant to Herceptin treatment 47. Our results showed that the dsRNA sensing pathway worked well in both parental and resistant cells, whereas the dsDNA sensing pathway was impaired in resistant cells. dsDNA could only promote the synthesis of cGAMP in parental cells but not in Herceptin-resistant cells. Treated with cGAMP or STINGa could reverse the inactivation of type I IFN signaling in resistant tumor cells, illustrating that the function of cGAS was broken. With Herceptin's selective pressure, tumor cells under stress could produce more fragments of nucleic acid entering cytoplasm, which would cause cell apoptosis, even without the involvement of immune pressure. Thus, tumor cells collectively choose to block their response to dsDNA. Multiple studies have showed that the enzymatic activity and DNA-binding ability of cGAS is subjected to modifications of the transcription level and various types of protein post-translational modifications, such as phosphorylation, ubiquitination, acetylation, glutamylation, and SUMOylation, which would suppress the activation of cGAS-STING pathway and subsequent type I IFN signaling 48, 49. The specific mechanisms underlying the impaired function of cGAS in Herceptin-resistant HER2+ BC require further investigation. Meanwhile, the antigen presentation capacity of cancer cells was impaired in Herceptin-resistant BC and could be restored by STINGa treatment. Interestingly, when resistant cells were co-cultured with PBMCs, ADU-S100 could increase anti-tumor effect. But when parental cells were co-cultured with PBMCs, ADU-S100 had no additional effect. These results were similar to several previous studies 31, 33. Jaclyn Sceneay *et al.* found that compared with young mice and young patients with triple-negative breast cancer, aged mice and aged patients showed decreased IFN signaling with age; STING agonist could significantly improve response to immune checkpoint blockade therapy in aged mice, but no additive effect of STING agonist in young mice, although STING agonist could also activate IFN signaling in young mice 33. Antonio Marzio *et al.* showed that mutations in *KEAP1* induces stabilization of EMSY, which was responsible for the suppression of IFN signaling and immune evasion in non-small cell lung cancer; STING agonist could inhibit the growth of *KEAP1*-mutant tumors, but not *KEAP1*-widetype tumors 31. It seems that tumors with defective IFN pathway displayed a sensitivity to STING agonism due to an engagement of anti-tumor immune signaling in the tumor microenvironment. If the tumor does not have a defective IFN pathway, it would not appear to be a vulnerability in this tumor type. The effects of ADU-S100 on anti-tumor immunity and tumor growth inhibition were further validated in the humanized mouse model bearing human Herceptin-resistant SKBR3 cells. Thus, these results suggested that the suppression of cGAS-STING pathway was the key factor in the immune escape of Herceptin-resistant tumor, and could be rescued by stimulating type I IFNs with STINGa.

Clinical trials have confirmed DS-8201 as the standard of care for HER2+ BC patients with Herceptin resistance 3. As a large proportion of Herceptin-resistant HER2+ BC patients are still refractory and not sensitive to HER2-targeting therapy, identifying novel combination strategies is a major priority 6. The mechanism of action of DS-8201 includes Antibody-dependent cell-mediated cytotoxicity (ADCC) and DNA damage induced by topoisomerase I inhibition. Since ADCC requires a suitable tumor immune microenvironment and DNA damage would exert anti-tumor effects by activating innate immunity, the suppressive immune microenvironment with impaired innate immune system may reduce the anti-tumor effect of DS-8201. We proposed that stimulating IFN signaling with STINGa may be a good strategy to compensate for defective innate immune signaling in Herceptin-resistant HER2+ BC and have a synergistic anti-tumor effect with DS-8201. Surprisingly, in the co-culture experiment with PBMCs, though no additional effect of ADU-S100 was observed in parental cells when combining with DS-8201, the combination of DS-8201 with ADU-S100 could increase the death of cancer cells significantly compared to DS-8201 alone in resistant cells *in vitro*. In humanized mouse model, the anti-tumor efficacy of combination of DS-8201 with ADU-S100 was significantly better than that of each single drug. Therefore, HER2+ BC with Herceptin resistance may benefit from a combination strategy including DS-8201 and IFN signaling activation with STINGa.

## Conclusion

In summary, we clarified a novel immune-related index (IRPI) for the prognosis of HER2+ BC patients with Herceptin treatment. We elucidated that cGAS-STING pathway is the key determinant of immune escape in Herceptin-resistant BC with high IRPI. And using STINGa in Herceptin-resistant HER2+ BC could reverse IFN signaling activity, which promotes anti-tumor immune response and have a synergistic anti-tumor effect with DS-8201 *in vitro* and *in vivo*.

## Supplementary Material

Supplementary figures and tables.
