# Supplementary material for: A High Immune-Related Index with the Suppression of cGAS-STING Pathway is a Key Determinant to Herceptin Resistance in HER2+ Breast Cancer
Source: Int J Biol Sci. 2024 Jun 17;20(9):3497–514. doi: 10.7150/ijbs.94868 (PMC11234227; doi:10.7150/ijbs.94868)
Supplement: Supplementary file 1 — Supplementary figures and tables. [file ijbsv20p3497s1.pdf]

Table S1: Key Resources

Antibodies	Source	Identifier	Dilution
IRF3	Proteintech	11312-1-AP-100UL	1:1000
HER2	Proteintech	18299-1-AP-50ul	1:1000
Phospho-IRF3 (Ser396)	Abcam	Ab138449	1:1000
cGAS	Proteintech	26416-1-AP-100ul	1:1000
TBK1	Cell signaling technology	38066S	1:1000
AKT1 (B-1)	Santa Cruz	sc-5298	1:500
Phospho-TBK1 (Ser172)	Cell signaling technology	5483S	1:1000
Phospho-AKT (Ser473)	Cell signaling technology	4060S	1:1000
GAPDH	Proteintech	60004-1-IG-100UL	1:5000
α-tubulin	Beyotime	AT819	1:5000
Anti-human HLA-A, B, C	BioLegend	311406	1:300
CD3	BioLegend	300313	1:500
CD28	BioLegend	302913	1:500
CD8a	Proteintech	66868-1-IG	1:300
GZMB	Proteintech	13588-1-AP	1:300
**Chemicals, peptides, and recombinant proteins**			
Lipofectamine 2000	Invitrogen	11668019	
HT-DNA	Sigma-Aldrich	D6898	
2’3’-cGAMP	Titan	04800926	
Poly(I:C)/dsRNA	InvivoGen	tlrl-picwlv	
Poly(dA:dT) / LyoVec	InvivoGen	tlrl-patc	
Human IL-2	PeproTech	200-02-100	
RIPA buffer	Cell signaling technology	9806s	
DAPI	Sigma-Aldrich	D9542	
ADU-S100	MCE	HY-12885B	
**Critical commercial assays**			
2’3’-cGAMP ELISA Kit	Cayman	501700	
RNA Purification Kit	EZBioscience		
HiScript II QRT SuperMix	Vazyme	R223-01	
ChamQ-SYBR-qPCR-MasterMix	Vazyme	Q311-02	
**qPCR Primers (human)**	**Forward (5’＞3’)**	**Reverse (5’＞3’)**	
IFNB1	GTCAGAGTGGAAATCCTAAG	TATGCAGTACATTAGCCATC	
GADPH	GATTCCACCCATGGCAAATTC	CTTCTCCATGGTGGTGAAGAC	
CCL5	AGCAGTCGTCTTTGTCAC	TAGCTCATCTCCAAAGAGTT	
CXCL10	TACCTGCATCAGCATTAGTA	TGTAGCAATGATCTCAACAC	

ISG15	GAACTCATCTTTGCCAGTA	ATCTTCTGGGTGATCTGC
IFIT-1	TACAGCAACCATGAGTACAA	TCACATAGGCTAGTAGGTTG
HLA-B	CAGTTCGTGAGGTTCGACAG	CAGCCGTACATGCTCTGGA
B2M	GAGGCTATCCAGCGTACTCCA	CGGCAGGCATACTCATCTTTT
TAPBP	TGGACCGGAAATGGGACCT	CCCCAGAAGGGTAGAAGTGG
TAP1	TGCCCCGCATATTCTCCCT	CACCTGCGTTTTCGCTCTTG
TAP2	TGGACGCGGCTTTACTGTG	GCAGCCCTCTTAGCTTTAGCA
ERAP1	CCCCTCAAATGGTCCCTTGC	GAGATGCTTCAGTGCTCTGAC

Table S2. Clinical characteristics of HER2+ breast cancer patients

Variables	Total patients n = 126 (%)	Nuclear-IRF3_high n = 63 (%)	Nuclear-IRF3_low n = 63 (%)	p-value
**Age**				0.35
**≤60**	104 (82.5)	50 (79.4)	54 (85.7)	
**>60**	22 (17.5)	13 (20.6)	9 (14.3)	
**Pathologic types**				0.72
**IDC**	118 (93.7)	60 (95.2)	58 (92.1)	
**DCIS**	8 (6.3)	3 (4.8)	5 (7.9)	
**Multifocality**				0.21
**Yes**	115 (91.3)	55 (87.3)	60 (95.2)	
**No**	11 (8.7)	8 (12.7)	3 (4.8)	
**Tumor location**				0.32
**Upper-inner**	22 (17.5)	10 (15.9)	12 (19.1)	
**Lower-inner**	6 (4.8)	2 (3.2)	4 (6.3)	
**Upper-outer**	70 (55.5)	35 (55.5)	35 (55.5)	
**Lower-outer**	18 (14.3)	8 (12.7)	10 (15.9)	
**Central**	10 (7.9)	8 (12.7)	2 (3.2)	
**T stage**				0.48
**T1**	30 (23.8)	14 (22.2)	16 (25.4)	
**T2**	85 (67.5)	42 (66.7)	43 (68.2)	
**T3**	8 (6.3)	6 (9.5)	2 (3.2)	
**T4**	3 (2.4)	1 (1.6)	2 (3.2)	
**N stage**				0.57
**N0**	59 (46.8)	30 (47.6)	29 (46.1)	
**N1**	26 (20.6)	13 (20.6)	13 (20.6)	
**N2**	21 (16.7)	8 (12.7)	13 (20.6)	
**N3**	20 (15.9)	12 (19.1)	8 (12.7)	
**M stage**				0.62
**M0**	122 (96.8)	60 (95.2)	62 (98.4)	
**M1**	4 (3.2)	3 (4.8)	1 (1.6)	
**Histological grade**				1.00
**I**	0 (0)	0 (0)	0 (0)	
**II**	64 (50.8)	32 (50.8)	32 (50.8)	
**III**	62 (49.2)	31 (49.2)	31 (49.2)	
**ER**				0.28
**Positive**	68 (54.0)	31 (49.2)	37 (58.7)	
**Negative**	58 (46.0)	32 (50.8)	26 (41.3)	
**PR**				1.00
**Positive**	54 (42.9)	27 (42.9)	27 (42.9)	
**Negative**	72 (57.1)	36 (57.1)	36 (57.1)	
**Ki-67 (%)**				0.85
**≤ 30**	81 (64.3)	41 (65.1)	40 (63.5)	
**> 30**	45 (35.7)	22 (34.9)	23 (36.5)	
**Endocrine therapy**				0.86
**Yes**	61 (48.4)	30 (47.6)	31 (49.2)	
**No**	65 (51.6)	33 (52.4)	32 (50.8)	
**Chemotherapy**				0.82
**Yes**	101 (80.2)	51 (81.0)	50 (79.4)	
**No**	25 (19.8)	12 (19.0)	13 (20.6)	

IDC: invasive ductal carcinoma; DCIS: ductal carcinoma in situ; ER: estrogen receptor; PR: progesterone receptor.

Figure S1

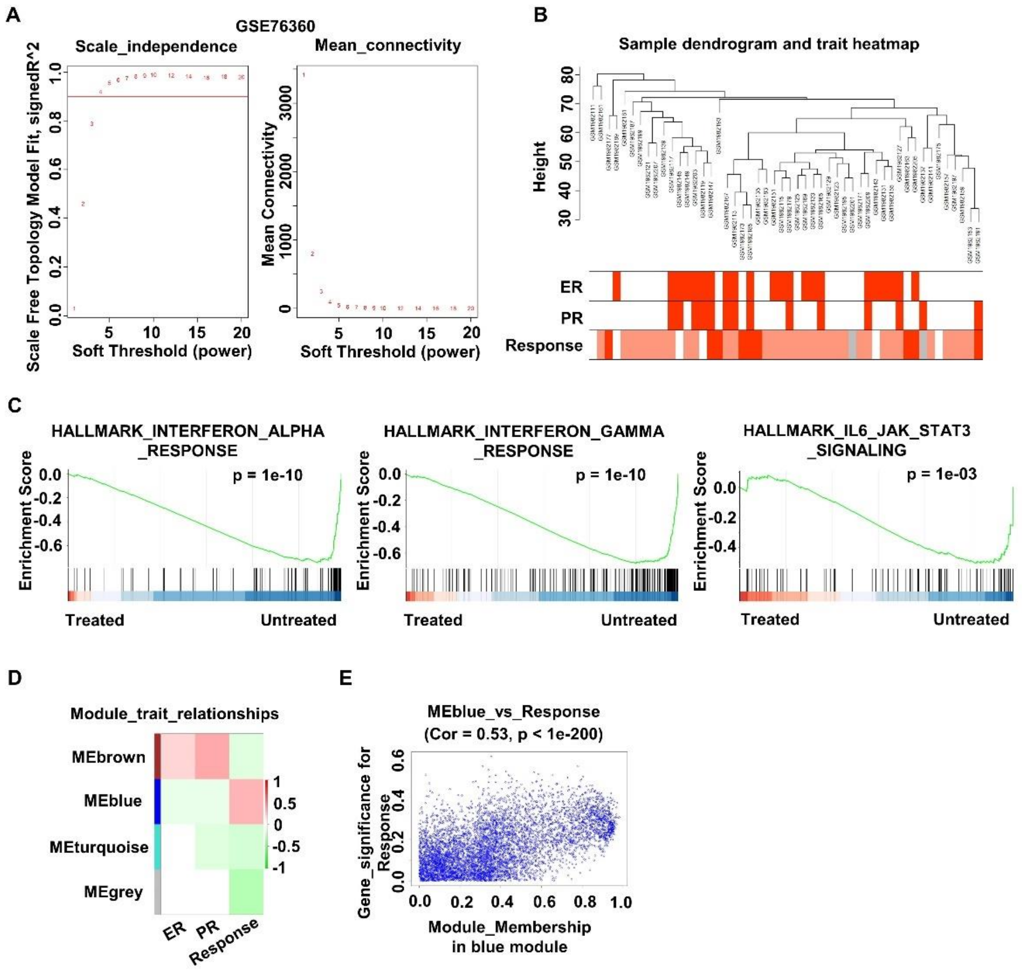
A
GSE76360
Scale_independence
Mean_connectivity
Scale Free Topology Model Fit, signedR^2
Mean Connectivity
Soft Threshold (power)
Soft Threshold (power)
B
Sample dendrogram and trait heatmap
Height
ER
PR
Response
C
HALLMARK_INTERFERON_ALPHA_RESPONSE
HALLMARK_INTERFERON_GAMMA_RESPONSE
HALLMARK_IL6_JAK_STAT3_SIGNALING
Enrichment Score
p = 1e-10
p = 1e-10
p = 1e-03
Treated
Untreated
D
Module_trait_relationships
MEbrown
MEblue
MEturquoise
MEgrey
ER
PR
Response
E
MEblue_vs_Response
(Cor = 0.53, p < 1e-200)
Gene_significance for Response
Module_Membership in blue module

Figure S2

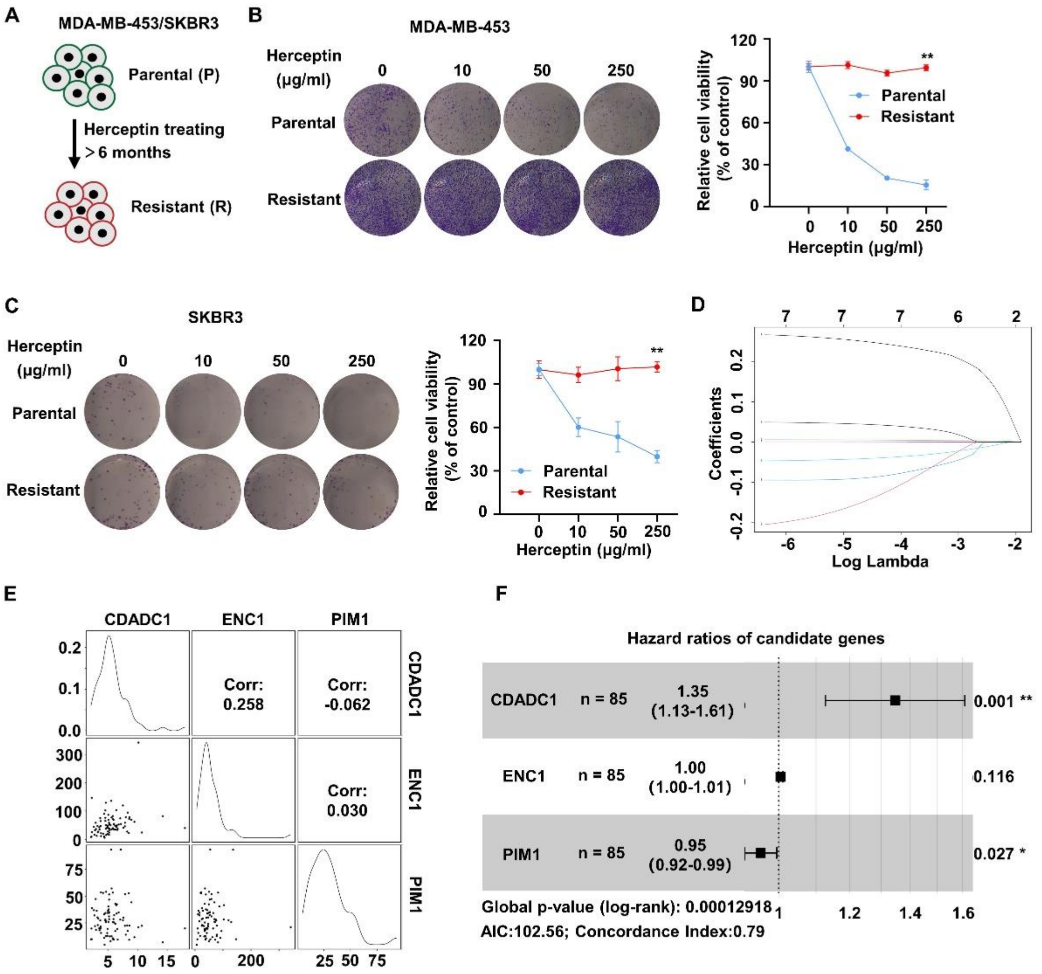

G

BANK1	FLT3LG	MMP16	SHANK3	APOC1	TMEM63C	SLC39A7	PIM1	MEF2C	THBD
BCL3	CPVL	PLA2G4C	GGA1	ACSL5	P2RX6P	CDKL1	AKR1C4	PCK1	MLXIPL
AKT3	ZNF14	PLA2G4F	RAB42	ZCCHC2	CAPG	CCDC78	AKAP1	DDX18	FOXA2
AUH	PLTP	FGFBP1	ERC2	D2HGDH	STARD5	EME2	PCP2	NAV2	OSBPL7
NPDC1	FAM110C	ZNF280C	CXorf56	TMOD2	FABP5	TTPA	HNF4A	MST1	EGLN3
CROT	HDX	TRPV6	NFE2L3	GIP	SORBS2	PRUNE2	BRSK1	CMTM3	ZFP36L1
ATP2B1	HSH2D	MC1R	ZNF177	NRCAM	ENC1	CDADC1	ALDH2	ZNF619	PLK2
RASA3	SLC25A34	CITED2	STAT2	IL6R	KCNMA1	GPR137C	MRPL30	C17orf67	TMPRSS5
KCNK10	GRIN2B	LPAL2							

Figure S3

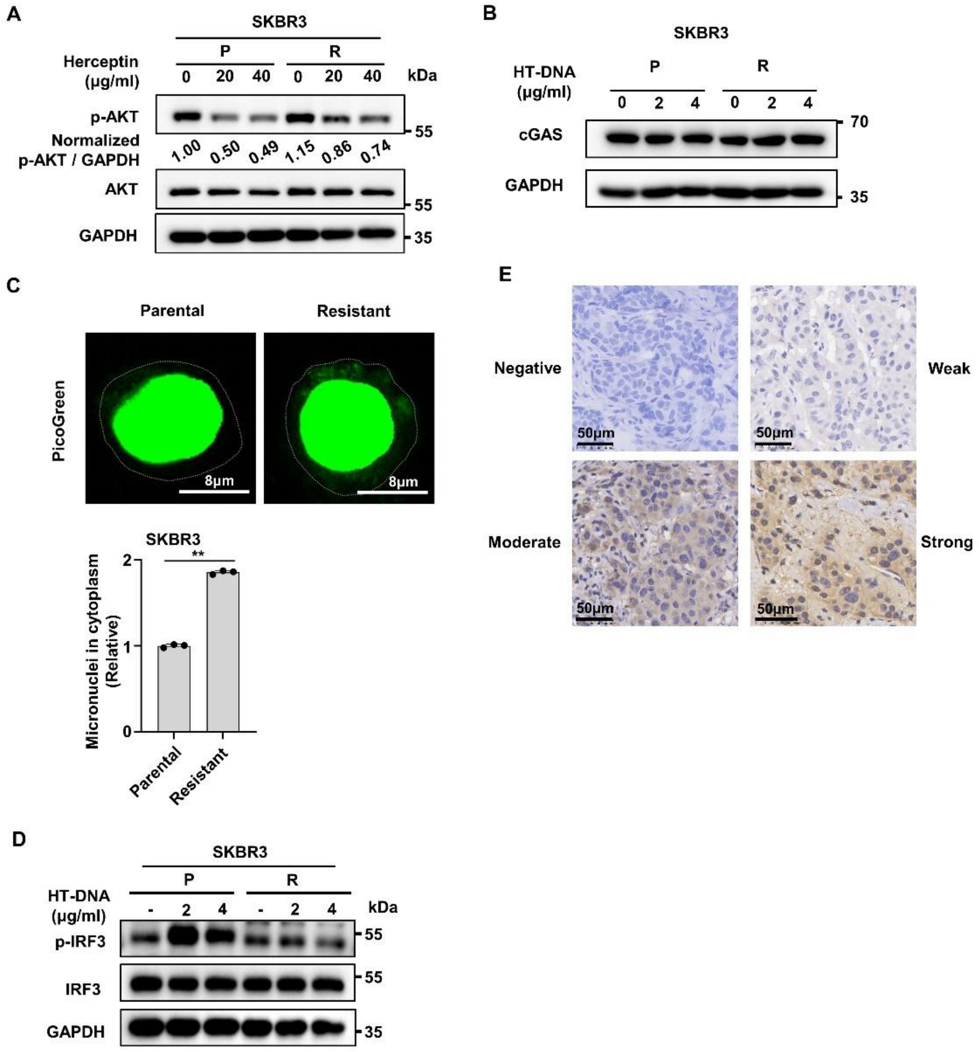
A
SKBR3
P
R
Herceptin (μg/ml)
0 20 40 0 20 40
kDa
p-AKT
55
Normalized p-AKT / GAPDH
1.00 0.50 0.49 1.15 0.86 0.74
AKT
55
GAPDH
35
B
SKBR3
HT-DNA (μg/ml)
P
R
0 2 4 0 2 4
cGAS
70
GAPDH
35
C
Parental
Resistant
PicoGreen
8μm
8μm
SKBR3
**
Micronuclei in cytoplasm (Relative)
0
1
2
Parental
Resistant
D
SKBR3
P
R
HT-DNA (μg/ml)
- 2 4 - 2 4
kDa
p-IRF3
55
IRF3
55
GAPDH
35
E
Negative
Weak
Moderate
Strong
50μm

Figure S4

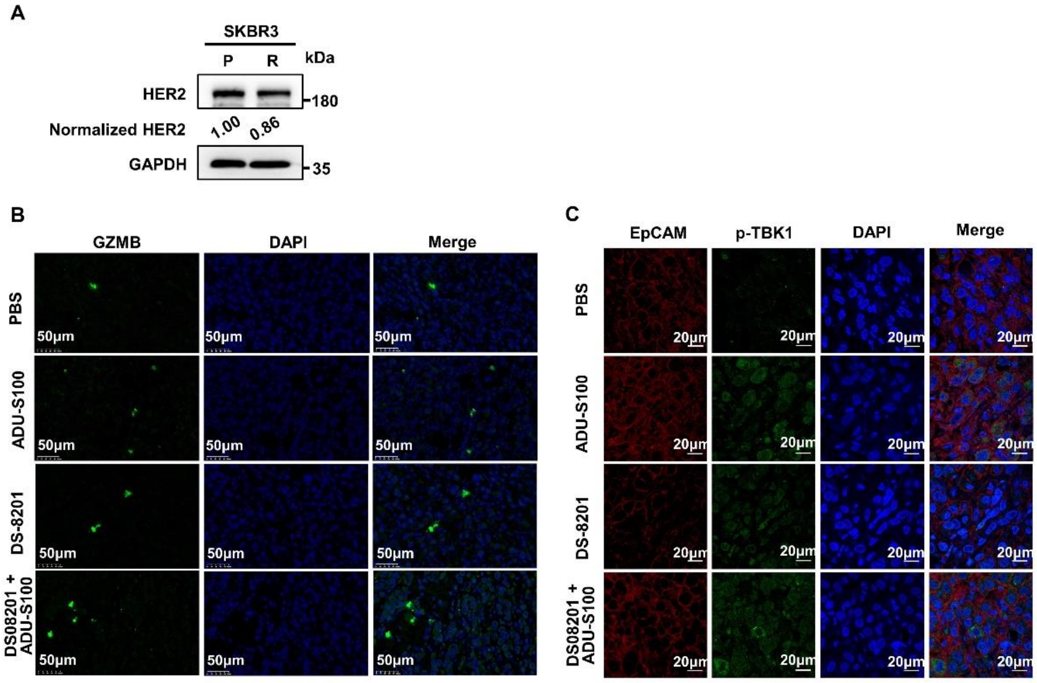
A
SKBR3
P
R
kDa
HER2
180
Normalized HER2
1.00
0.86
GAPDH
35
B
GZMB
DAPI
Merge
PBS
ADU-S100
DS-8201
DS08201 + ADU-S100
50μm
C
EpCAM
p-TBK1
DAPI
Merge
PBS
ADU-S100
DS-8201
DS08201 + ADU-S100
20μm
